# 

**Published:** 1847-04

**Authors:** 


### WESTERN JOURNAL

### OF

### MEDICINE AND SURGERY.

### EDITORS.

---

DANIEL DRAKE, M.D.,

PROFESSOR OF PATHOLOGY AND THE PRACTICE OF MEDICINE IN THE MEDICAL INSTITUTE OF LOUISVILLE:

LUNSFORD P. YANDELL, M.D.,

PROFESSOR OF CHEMISTRY AND PHARMACY IN THE SAME:

THOMAS W. COLESCOTT, M.D.

### BOOKS RECEIVED.

Through Messrs. Maxwell & Prescott, booksellers of this city, in addition to the books mentioned in another department of the Journal, we have received the following works: Wilson's Anatomy, third edition, Von Behr's Hand-book of Human Anatomy, Philosophy in Sport made Science in Earnest, and a supplementary volume to the popular Encyclopædia Americana.

